# Improving the Response of Accelerometers for Automotive Applications by Using LMS Adaptive Filters

**DOI:** 10.3390/s100100313

**Published:** 2009-12-31

**Authors:** Wilmar Hernandez, Jesús de Vicente, Oleg Sergiyenko, Eduardo Fernández

**Affiliations:** 1 Department of Circuits and Systems, EUIT de Telecomunicación, Universidad Politécnica de Madrid (UPM), Campus Sur UPM, Ctra. Valencia km 7, Madrid 28031, Spain; 2 Department of Applied Physics, ETSI Industriales, Universidad Politécnica de Madrid, Calle José Gutierrez Abascal 2, Madrid 28006, Spain; E-Mail: jvicente@etsii.upm.es; Tel.: +34913363125; Fax: +34913363000; 3 Institute of Engineering, Autonomous University of Baja California, Mexicali, Baja California, Mexico; E-Mail: srgnk@iing.mxl.uabc.mx; 4 EUIT de Telecomunicación, Universidad Politécnica de Madrid (UPM), Campus Sur UPM, Ctra. Valencia km 7, Madrid 28031, Spain; E-Mail: edfernan@alumnos.euitt.upm.es

**Keywords:** piezoresistive accelerometer, 4-order band-pass digital Butterworth filter, LMS adaptive filter

## Abstract

In this paper, the least-mean-squares (LMS) algorithm was used to eliminate noise corrupting the important information coming from a piezoresisitive accelerometer for automotive applications. This kind of accelerometer is designed to be easily mounted in hard to reach places on vehicles under test, and they usually feature ranges from 50 to 2,000 g (where is the gravitational acceleration, 9.81 m/s^2^) and frequency responses to 3,000 Hz or higher, with DC response, durable cables, reliable performance and relatively low cost. However, here we show that the response of the sensor under test had a lot of noise and we carried out the signal processing stage by using both conventional and optimal adaptive filtering. Usually, designers have to build their specific analog and digital signal processing circuits, and this fact increases considerably the cost of the entire sensor system and the results are not always satisfactory, because the relevant signal is sometimes buried in a broad-band noise background where the unwanted information and the relevant signal sometimes share a very similar frequency band. Thus, in order to deal with this problem, here we used the LMS adaptive filtering algorithm and compare it with others based on the kind of filters that are typically used for automotive applications. The experimental results are satisfactory.

## 1. Introduction

When designing a sensor system, one of the most difficult parts is to carry out high quality filtering of any unwanted information. In practice, we cannot eliminate totally this unwanted information, but what we can do is to make use of the advances in technology to build intelligent sensor systems able to diminish the noise corrupting the relevant information coming from sensors down to noise levels at which their negative effect on the important signal is negligible. Recent applications of several advanced filtering techniques have shown that the signal-to-noise ratio (SNR) can be increased by using appropriate filtering techniques [1–23].

In this context, in the scientific literature there is wide range of filtering algorithms to be implemented by using either analog electronics or digital one; and such algorithms can be either optimal with respect to some index of performance or robust with respect to structured and unstructured uncertainties [24,25] or neither optimal nor robust.

Very often, when designing sensor systems, designers tend to build the signal treatment stages using the classical approach to filtering [26,27] and signal conditioning [28,29]. In addition, such systems are custom-built to perform satisfactorily under certain, very specific working conditions, in environments in which we know the noise characteristics, the frequency of the important signal, the operating temperature, and other environmental conditions. Thus, sensor manufactures try to develop products that meet their customer’s needs.

However, the above statement also brings about two problems. First, as a custom-built sensor system is designed to solve only one specific problem with some constraints, if the working conditions change, the system is not adapted to deal with those changes. Second, custom-built sensor systems are far from being inexpensive. Therefore, both the cost and the ability of the system for adapting itself to new, unpredictable changes and for adjusting its own parameters automatically in an active interaction with the environment are of paramount importance.

For this reason, in this paper we present a comparative analysis between the results of the traditional way of filtering and the ones of using easy, inexpensive adaptive filtering [30,31] to improve the performance of the piezoresistive accelerometer 1201F of the manufacturer Measurement Specialties. Here we used the least-mean-squares (LMS) adaptive filtering algorithm to carry out the optimal filtering process.

## 2. The Accelerometer

The principles of accelerometers are described in several references on sensors and actuators [32], and there is a wide variety of accelerometers that could be used in various applications depending on the requirements of range, natural frequency, damping, temperature, size, weight, hysteresis, low noise, and so on. Piezoelectric accelerometers, piezoresistive accelerometers, variable capacitance accelerometers, linear variable differential transformers (LVDT), variable reluctance accelerometers, potentiometric accelerometers, gyroscopes used for sensing acceleration, strain gauges accelerometers, among others, are some examples of the types of accelerometers that exist [28,29,32].

**Figure 1.**
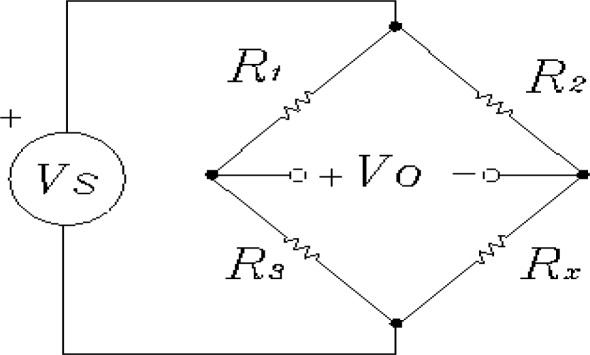
The Wheatstone bridge circuit.

In this paper, we are interested in measuring steady-state accelerations and the DC accelerometer 1,201 F of Measurement Specialties was tested under laboratory conditions for future use in automotive applications. Basically, the schematic diagram of this accelerometer consists of a configuration of the well-known Wheatstone bridge circuit like the one shown in Figure 1, which can be a one-arm, a two-arms or a four-arms bridge configuration. In this figure, *V_S_* represents the excitation (2–10 VDC excitation for maximum flexibility), *V_0_* is the output voltage, and *R*_1_, *R*_2_, *R*_3_, and *R_x_* are one (*i.e.*, one-arm bridge configuration), two (*i.e.*, two-arms bridge configuration) or four (*i.e.*, four-arms bridge configuration) resistors whose resistance depend on the acceleration. The Wheatstone bridge circuit is a very well known one and information about how to obtain the bridge off-null voltage can be found in many references, for example in [11,28,29,32], among others.

The features of the 1,201 F accelerometer are the following: 2nd generation MEMS sensing element; 1,000 g Full Scale Range; 2–10 VDC Excitation for Maximum Flexibility; 0–50 °C Temperature Compensation; ± 40 mV Zero Measurand Output; Gas Damping; Connector Options; Mechanical Overload Stops; and Designed for Screw Mounting. In addition, its applications are the following: Crash Testing, Impact Testing; Off-Road Testing; and Road Testing. More information about the model 1201F accelerometer can be found on the website of the manufacturer: www.meas-spec.com.

## 3. Conventional and Optimal Adaptive Filtering

In spite of the fact that sensor manufacturers are working hard to adapt processes used to manufacture advanced semiconductor technologies to the manufacturing of sensors, and improve the performance of sensors by using integrated circuit technologies [33], most of the algorithms that smart sensors use to carry out the filtering of unwanted signals are based on classical filtering techniques.

For instance, according to [34], the practical accelerometer analog interface circuit design of airbags usually has a low-pass filter that is a 2- or 4-pole Bessel function that is unable to cancel satisfactorily the signal that corrupts the relevant information coming from the accelerometer. This is because both the bandwidth of the real-time relevant signal and noise characteristics are unknown, and at low excitation levels the SNR is so small that the electronic system can confuse noise with relevant signal information and activate the airbag when it is not needed, which is a safety related problem.

Then, in order to prevent the system from activating the airbag when the excitation is below certain levels, other electronic circuits are used. Thus, the filtering problem does not rely completely on the low-pass filter they use.

Therefore, in practical accelerometer architectures, in order to avoid that the output be a false representation of the original signal, the signal is redistributed. Nevertheless, this redistribution of gain requires knowledge of the worst-case signals to be applied and an acceptance of noise in the output signal [34].

Taking into consideration the above statements it cannot be said that using conventional filters is the best option we have to develop a solution that meets the performance objectives. To be more specific, as mentioned in [34], there are cases in which the low-pass filter cannot suppress the noise and attenuates the relevant signal, causing serious distortions that affect the performance of crash-detection algorithms and decrease the SNR at the output of the sensing system. This problem is a safety-related problem that deserves our full attention.

**Figure 2.**
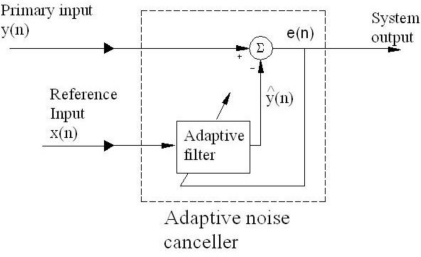
Schematic diagram of the ANC.

On the other hand, one of the advantages of adaptive filters is that they have a mechanism for adjusting its own parameters automatically by using a recursive algorithm, at the same time that the filter is in active interaction with the environment. Therefore, they can perform satisfactorily in environments in which we have little knowledge of the noise characteristics, and the SNR improvement achieved with these filters is several times better than the one achieved by using the conventional ones [13]. Furthermore, another very important advantage of using some adaptive filtering algorithms is its simplicity. In this paper, we use the LMS adaptive filter and show its benefits over conventional filters. To that end, here we are going to use an adaptive noise canceller (ANC) device [30,31] based on the conventional LMS adaptive filter algorithm. Figure 2 shows the schematic diagram of such a device.

The practical implementation of the LMS algorithm is very simple and it is well documented in [30,31], among other highly regarded international references. In accordance with [30,31], the steps of the implementation of the LMS algorithm are the following:

First, the *output signal* of the adaptive filter:

$$\hat{y}\left(n\right)={\hat{W}}^{H}\;\left(n\right)x\left(n\right)$$

where the superscript *H* denotes *Hermitian transposition*, is obtained. This signal is the scalar product of the tap-weight vector of the filter **Ŵ**(*n*) (with length *M*) and the tap-input vector **x**(*n*) (with length *M*). From Figure 2, it can be seen that the input vector **x**(*n*) is given by:

$$x\left(n\right)={\left[\begin{array}{lll} x\left(n\right) & \cdots & x\left(n-M+1\right) \end{array}\right]}^{T}$$

where the superscript *T* denotes *transposition* and *x*(*n*) is the *reference* (*auxiliary*) *input* to the filter.

Second, the *estimation error* (or system output in Figure 2):

$$e\left(n\right)=y\left(n\right)-\hat{y}\left(n\right)$$

which is the difference between the *desired response* (or *primary input*) *y*(*n*) and the output signal, is obtained.

Third, the conjugate error signal, the tap-input vector, the tap-weight vector and the (constant) step-size parameter *μ*, all of them at the iteration *n*, are used to obtain the tap-weight vector for the next iteration *n* + 1. That is, the *tap-weight adaptation* is given by:

$$\hat{W}\left(n+1\right)=\hat{W}\left(n\right)+\mu \cdot x\left(n\right)\cdot e^{*}\left(n\right)$$

Then, repeat all the steps again starting from the first one for *N* iterations, starting from *n* = 0 with the initial condition of the tap-weight vector **Ŵ**(0).

## 4. Results of the Experiment

In the experiment, the accelerometer 1201F-1000-10-240X (Model 1201F, 1,000 g Full Scale Range, 10 VDC excitation, 240 inches cable, and no options), was tested under laboratory conditions by using the calibration system CS18 TF from SPEKTRA. This system can carry out calibrations of sensors with/without amplifiers in the frequency range 3 Hz to 5 kHz, with a repeatability of the calibration under identical conditions up to 5 kHz better than 0.5%.

Here, the 1201F-1000-10-240X accelerometer was tested at 50 Hz, 100 Hz, 200 Hz, 500 Hz and 1 kHz, with a sinusoidal acceleration excitation of amplitude 2 g. Furthermore, the National Instruments Data Acquisition Card NI DAQCard-6062E was used for the laboratory experiments. In addition, for the experiments at 50 Hz and 100 Hz the sampling frequency was 30 kHz, and for the experiments at 200 Hz, 500 Hz and 1 kHz the sampling frequency was 100 kHz.

**Figure 3.**
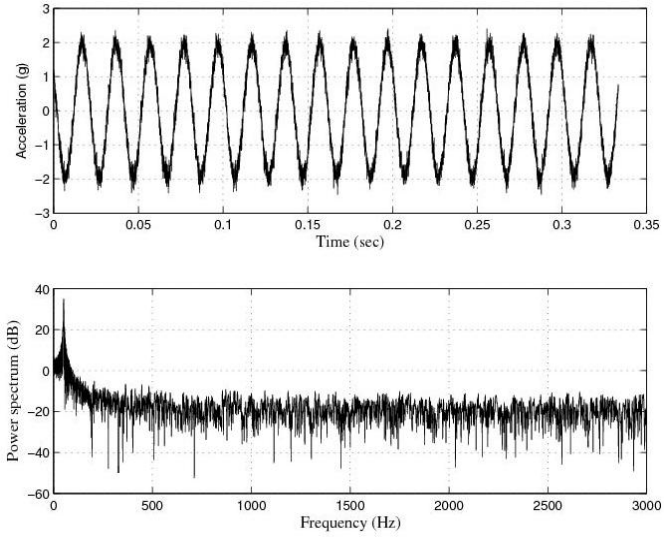
Response of the sensor system before filtering for a sinusoidal excitation of 2 g of amplitude at 50 Hz: Acceleration (or output signal) (g) and Power spectrum magnitude (dB).

**Figure 4.**
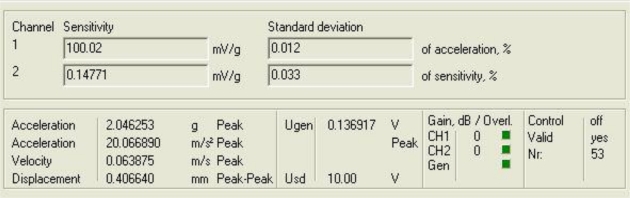
Current values of the calibration run: 50 Hz.

Figure 3 shows the response of the sensor system before filtering for the above excitation at 50 Hz, and Figure 4 shows a diagram window with the current values of the calibration run. In that diagram window it is shown the sensitivity if the reference sensor (Channel 1 Sensitivity) and the currently measured sensitivity of the sensors under test (Channel 2 Sensitivity). In addition, the current Standard deviations of acceleration and sensitivity of the sensor under test, the instantaneous values of Acceleration, Velocity and Displacement of the vibration exciter, the Generator voltage (output signal) and the selected Gain are read out in the boxes named accordingly. Control indicates whether control is enabled or disabled.

Finally, Overload Channel 1 or 2 (red) indicates that the input voltage of the AD converter exceeds the permissible maximum value, Overload Generator (red) indicates that the controller is unable to establish the required amplitude, and box Valid is for indicating whether the result is valid (target acceleration established).

**Figure 5.**
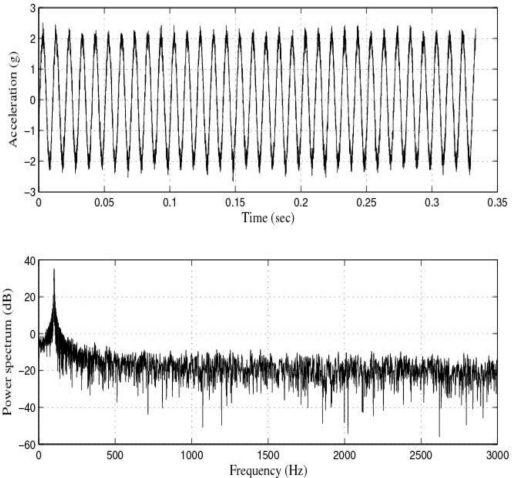
Response of the sensor system before filtering for a sinusoidal excitation of 2 g of amplitude at 100 Hz: Acceleration (or output signal) (g) and Power spectrum magnitude (dB).

**Figure 6.**
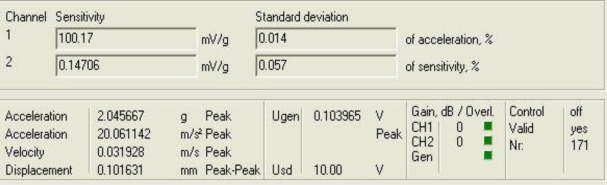
Current values of the calibration run: 100 Hz.

Figures 5 and 6 show the response of the sensor system before filtering for the above excitation at 100 Hz and a diagram window with the current values of the calibration run.

**Figure 7.**
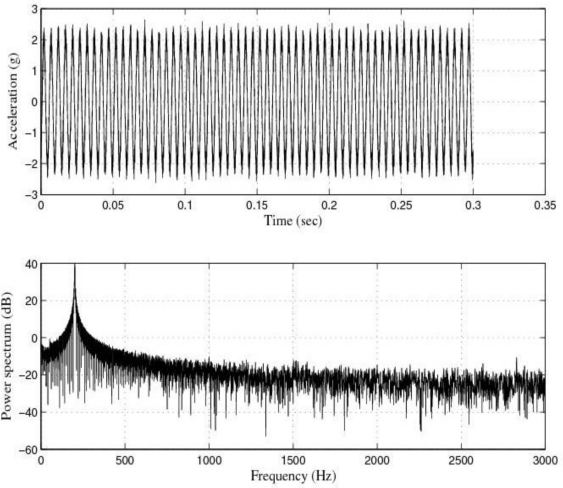
Response of the sensor system before filtering for a sinusoidal excitation of 2 g of amplitude at 200 Hz: Acceleration (or output signal) (g) and Power spectrum magnitude (dB).

**Figure 8.**
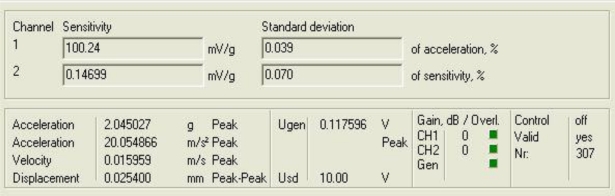
Current values of the calibration run: 200 Hz.

Figures 7 and 8 show the response of the sensor system before filtering for the above excitation at 200 Hz and a diagram window with the current values of the calibration run.

**Figure 9.**
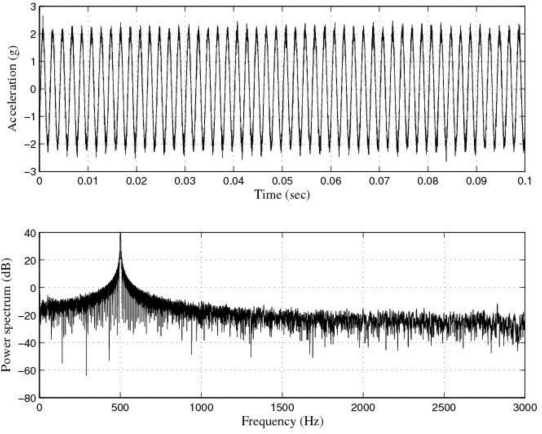
Response of the sensor system before filtering for a sinusoidal excitation of 2 g of amplitude at 500 Hz: Acceleration (or output signal) (g) and Power spectrum magnitude (dB).

**Figure 10.**
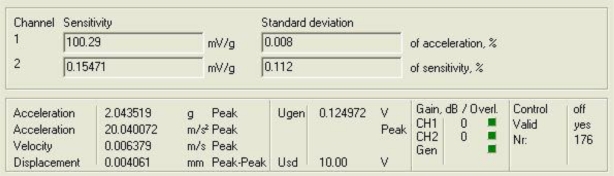
Current values of the calibration run: 500 Hz.

Figures 9 and 10 show the response of the sensor system before filtering for the above excitation at 500 Hz and a diagram window with the current values of the calibration run.

**Figure 11.**
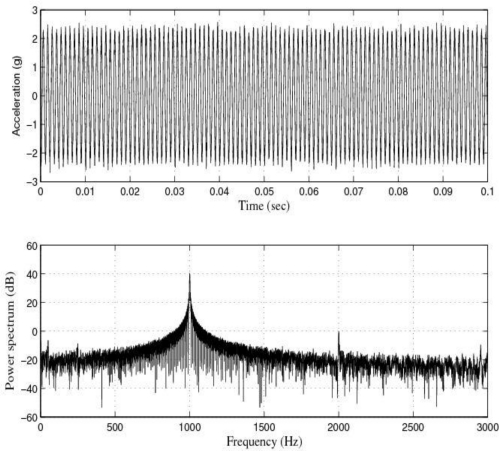
Response of the sensor system before filtering for a sinusoidal excitation of 2 g of amplitude at 1 kHz: Acceleration (or output signal) (g) and Power spectrum magnitude (dB).

**Figure 12.**
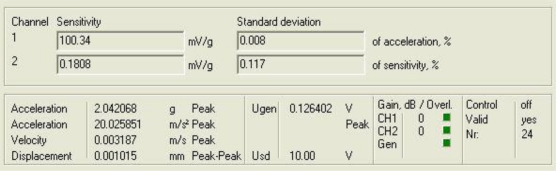
Current values of the calibration run: 1,000 Hz.

Figures 11 and 12 show the response of the sensor system before filtering for the above excitation at 1 kHz and a diagram window with the current values of the calibration run.

The response of the sensor to the above excitations shown in Figures 3, 5, 7, 9 and 10 indicate that it was necessary to cancel the noise corrupting the relevant signal. To that end, the first thing we did was to filter the signal coming from the sensor by using the kind of filters currently used in today’s automotive systems. That is, by using conventional filters.

Thus, we had two options: the first one was to use a low-pass filter with *cut-off frequency* at the frequency of the sinusoidal acceleration excitation.

The second one was to use a band-pass filter with *center frequency* at the frequency of the sinusoidal acceleration excitation.

**Figure 13.**
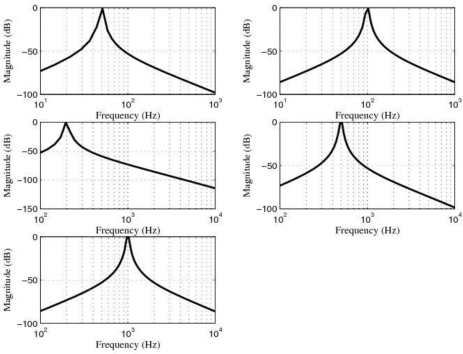
Magnitude (dB) of the frequency response of the five 4-order band-pass digital Butterworth filters.

**Figure 14.**
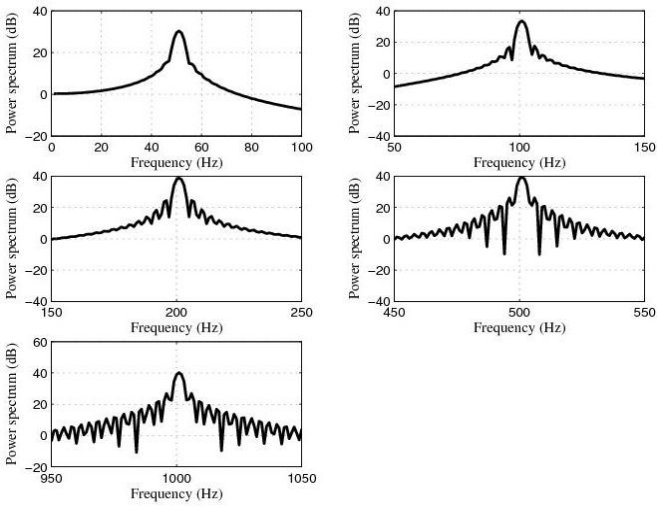
Power spectrum magnitude (dB) of the output signals after filtering by using the corresponding five 4-order band-pass digital Butterworth filters.

However, in spite of the fact that the first option was not a bad idea, we would have the problem of allowing low-frequency noise and disturbances to pass through the sensor system. For this reason, that option was discarded. Therefore, in order to accomplish the task of filtering, we used five 4-order band-pass digital Butterworth filters with center frequencies at 50 Hz, 100 Hz, 200 Hz, 500 Hz and 1 kHz, respectively. Furthermore, in order to follow the same design criterion, the *quality factor* Q of all of these filters was equal to 20. Q could have been chosen to be greater than 20 but 20 was a reasonable choice. The approximate system functions of these filters are shown in Table 1, the approximate locations of their zeros and poles are shown in Table 2, the magnitude of the frequency response of these filters is shown in Figure 13 and the power spectrum of the output signals, after filtering, are shown in Figure 14.

At this point, it is important to mention that the performance of the sensor system based on the five 4-order band-pass digital Butterworth filters can be considered satisfactory. However, in real automotive applications the designer does not know exactly the frequency of the relevant signal.

Therefore, the designer cannot design a bank of band-pass filters to cope with the noise/disturbance rejection problem, because he/she does not know what are the center frequencies of his/her filters. What is more, even in the case of knowing the frequencies of the excitation signals, if there were several of them, then the bank of filters would consist of several filters. Such a bank of filter would be expensive if it were implemented by using analog electronics, and would have problems due to numerical properties of the filters if it were implemented by using digital electronics.

Thus, in order to diminish the noise that corrupts the relevant signal coming from the sensors in a more efficient and cheaper manner, we used an adaptive filter. A filter that placed in an ANC device (see Figure 2) can perform as an entire bank of band-pass filters and that can adjust automatically its center frequency by itself, without needing any human intervention.

In this sense, as automotive applications require robust, easy to implement devices, because they have to work for long periods of time and make decisions in situations that involve safety-related problems, for the case under study we solved the noise rejection problem by using an LMS adaptive filter.

The parameters of the LMS adaptive filter (see Section 3) were the following: a tap-weight vector of length *M* equal to 100, and a step-size parameter *μ* equal to 1 over the maximum value of the power of the tap-input vector **x**(*n*) [31].

**Figure 15.**
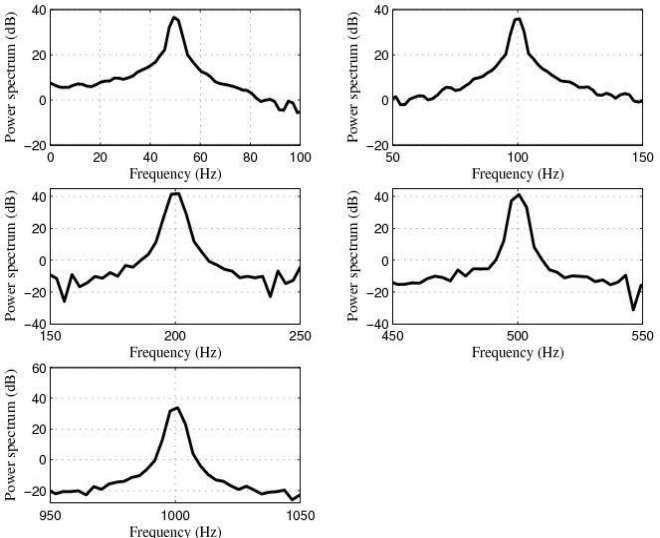
Power spectrum magnitude (dB) of the output signals after filtering by using the LMS adaptive filter.

**Figure 16.**
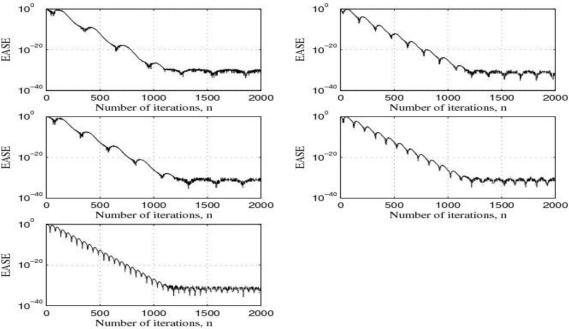
Learning curves of the LMS adaptive filter for the cases under test: EASE is the ensemble-average squared error (logarithmic scale).

**Figure 17.**
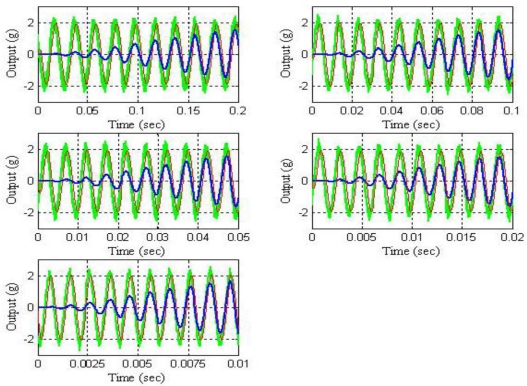
Time waveforms of the output signal for the five cases under test: Green—output signal (g) before filtering; Blue—output signal (g) after filtering by using the 4-order band-pass digital Butterworth filters; and Red—output signal (g) after filtering by using the LMS adaptive filter.

Figure 15 shows the power spectrum of the output signals after filtering by using the LMS adaptive filter and Figure 16 shows the learning curves of the LMS adaptive filter for the five cases under test. Also, Figure 17 shows the time waveforms of the output signals before filtering and after filtering by using both the 4-order band-pass digital Butterworth filters and the LMS adaptive filter.

If we compare the experimental results shown in Figure 15 with the ones shown Figure 14 and analyze the results shown in Figure 17, we can see that both the *quality of the response* and the *speed of the response* of the LMS adaptive filter are better than the ones of the five 4-order band-pass digital Butterworth filters. Therefore, the best option to carry out the filtering problem discussed in this paper was to use the LMS adaptive filter.

## 5. Conclusions

In this paper, an LMS adaptive filter was used to cancel the noise that corrupts the relevant information coming from an accelerometer under laboratory tests. The results of the experiment were satisfactory. Also, in order to show that the performance of the LMS adaptive filter was better than the one of the kind of filters used for automotive applications, the adaptive filter was compared with five 4-order band-pass digital Butterworth filters. The results of the experiment showed that the adaptive filter was superior to the band-pass digital Butterworth filters.

## Figures and Tables

**Table 1.** Approximate system functions of the five 4-order band-pass digital Butterworth filters.

	**System functions**
*H*_50_(*z*)	$\frac{1.34\times 10^{-7}-2.74\times 10^{-7}z^{-2}+1.37\times 10^{-7}z^{-4}}{1-3.9987z^{-1}+5.9962z^{-2}-3.9966z^{-3}+9.9895\times 10^{-1}z^{-4}}$
*H*_100_ (*z*)	$\frac{5.48\times 10^{-7}-10.95\times 10^{-7}z^{-2}+5.48\times 10^{-7}z^{-4}}{1-3.9970z^{-1}+5.9920z^{-2}-3.9928z^{-3}+9.9791\times 10^{-1}z^{-4}}$
*H*_200_ (*z*)	$\frac{1.97\times 10^{-7}-3.95\times 10^{-7}z^{-2}+1.97\times 10^{-7}z^{-4}}{1-3.9984z^{-1}+5.9956z^{-2}-3.9959z^{-3}+9.9874\times 10^{-1}z^{-4}}$
*H*_500_ (*z*)	$\frac{1.23\times 10^{-6}-2.46\times 10^{-6}z^{-2}+1.23\times 10^{-6}z^{-4}}{1-3.9949z^{-1}+5.9866z^{-2}-3.9886z^{-3}+9.9686\times 10^{-1}z^{-4}}$
*H*_1*k*_ (*z*)	$\frac{4.92\times 10^{-6}-9.84\times 10^{-6}z^{-2}+4.92\times 10^{-6}z^{-4}}{1-3.9858z^{-1}+5.9655z^{-2}-3.9733z^{-3}+9.9374\times 10^{-1}z^{-4}}$

**Table 2.** Approximate locations of the zeros and poles of the five 4-order band-pass digital Butterworth filters.

	**Zeros**	**Poles**
*H*_50_ (*z*)	1 ± j·1.710747438529511·10^−8^ −9.999999935682686·10^−1^ −1.000000006431731	9.996741670997507·10^−1^ ± j·1.072413775639032·10^−2^ 9.996927386471830·10^−1^ ± j·1.020085913032332·10^−2^
*H*_100_ (*z*)	1 ± j·1.710747438529511·10^−8^ −9.999999935682686·10^−1^ −1.000000006431731	9.992334342881192·10^−1^ ± j·2.144130574975457·10^−2^ 9.992815130644069·10^−1^ ± j·2.039543280194388·10^−2^
*H*_200_ (*z*)	1 ± j·1.710747438529511·10^−8^ −9.999999935682686·10^−1^ −1.000000006431731	9.995952091041387·10^−1^ ± j·1.286817863878919·10^−2^ 9.996188077792785·10^−1^ ± j·1.224029964677269·10^−2^
*H*_500_ (*z*)	1 ± j·1.710747438529511·10^−8^ −9.999999935682686·10^−1^ −1.000000006431731	9.986779155639293·10^−1^ ± j·3.215024955884750·10^−2^ 9.987664173461175·10^−1^ ± j·3.058269112594961·10^−2^
*H*_1*k*_ (*z*)	1 ± j·1.710747438529511·10^−8^ −9.999999935682686·10^−1^ −1.000000006431731	9.963239762599312·10^−1^ ± j·6.421553359261849·10^−2^ 9.965990147634185·10^−1^ ± j·6.108996200907289·10^−2^
